# Experiments Made to Determine the Positive and Relative Quantities of Moisture Absorbed from the Atmosphere by Various Substances, under Similar Circumstances

**Published:** 1788

**Authors:** Benjamin Thompson


VII. *Experiments made to determine the positive and relative Quantities of Moisture absorbed from the Atmosphere by various Substances, under similar Circumstances. By Sir* Benjamin Thompson, *Knt. F. R. S.* Vide *Philosophical Transactions of the Royal Society of London.* Vol. LXXVII. *For the Year* 1787. Part II. 4to. London, 1787.

As these experiments relate particularly to such substances as are commonly made use of for cloathing, an account of them cannot fail of being interesting to the medical reader.

The author having procured a quantity of the undermentioned substances, in a state of the most perfect cleanness and purity, exposed them, spread out upon clean china-plates, twenty-four hours in the dry air of a very warm room (which had been heated every day for several months by a German stove), the heat, during the last six hours, having been kept up to 85° of Fahrenheit's thermometer; after which he weighed equal quantities of these various substances in the room, with a very accurate balance, as expressed in the following table.

The same substances were then removed into into a large uninhabited room on the ſecond floor, and there expoſed, in the ſame manner as before, during the ſpace of forty-eight hours, on a table placed in the middle of the room, the air of the room being at the temperature of 45°; after which they were again carefully weighed in the room, and found to weigh as under mentioned.

They were next removed into a very damp cellar, and placed upon a table, in the middle of a vault, where the air, which appeared by the hygrometer to be completely ſaturated with moiſture, was at the temperature of 45°; and in this ſituation they were ſuffered to remain three days and three nights, the vault being hung round, during all this time, with wet linen cloths, to render the air as damp as poſſible, and the door of the vault being ſhut.

At the end of the three days, each ſubſtance was weighed by the Author upon the ſpot, and found to weigh as is expreſſed in the third column of the following table.

| The various fubftances | Wgt. after beingdried 24 hours in a hot room. | Wgt. after being ex-pofed 48 hours in a cold, un-inhabited room. | Wgt. after being ex-pofed 72 hours in a damp cel-lar. |
|---|---|---|---|
| | Pts. | Pts. | Pts. |
| Sheep's wool — — | 1000 | 1084 | 1163 |
| Beaver's fur — — | 1000 | 1072 | 1125 |
| The fur of a Ruffian hare — | 1000 | 1065 | 1115 |
| Eider down — — | 1000 | 1067 | 1112 |
| Silk { Raw, fingle thread | 1000 | 1057 | 1107 |
| Silk { Ravelings of white taffety | 1000 | 1054 | 1103 |
| Linen { Fine lint — | 1000 | 1046 | 1102 |
| Linen { Ravelings of fine linen | 1000 | 1044 | 1082 |
| Cotton wool — — | 1000 | 1043 | 1089 |
| Silver wire, very fine, gilt, and flatted, being the ravelings of gold lace — — | 1000 | 1000 | 1000 |

The weight made ufe of in thefe experiments was that of Cologne, the parts or leaft divifions being $=\frac{1}{65536}$ part of a mark, confequently 1000 of thefe parts make about $52\frac{3}{4}$ grains Troy.

The Author obferves that he did not add the filver wire to the other fubftances from any idea that it could poffibly imbibe moifture from the atmofphere; but merely to fee whether a metal, placed in air faturated with water, is not capable of receiving a fmall ad-

 dition dition of weight from the moiſture attracted by it, and attached to its ſurface. From the reſult of the experiment, however, he remarks, it would ſeem that no ſuch attraction ſubſiſts between the metal he employed, and the watery vapour diſſolved in air.

From the above table it ſhould ſeem that thoſe bodies which are the moſt eaſily wet, or which receive water in its unelaſtic form, with the greateſt eaſe, are not thoſe which in all caſes attract the watery vapour diſſolved in the air with the greateſt force.

Perhaps, remarks the Author, the apparent dampneſs of linen, to the touch, ariſes more from the eaſe with which that ſubſtance parts with the water it contains, than from the quantity of water it actually holds : in the ſame manner as a body appears hot to the touch, in conſequence of its parting freely with its heat, while another body, which is actually at the ſame temperature, but which witholds its heat with greater obſtinacy, affects the ſenſe of feeling much leſs violently.

It is well known, he obſerves, that woollen clothes, ſuch as flannels, &c. worn next the ſkin, greatly promote inſenſible perſpiration ; and may not, he aſks, this ariſe principally from from the ſtrong attraction which ſubſiſts between wool and the watery vapour that is continually iſſuing from the human body?

That it does not depend entirely upon the warmth of that covering, is, he thinks, clear; for the ſame degree of warmth, produced by wearing more cloathing of a different kind, does not produce the ſame effect.

He obſerves that the perſpiration of the human body being abſorbed by a covering of flannel, it is immediately diſtributed through the whole thickneſs of that ſubſtance, and by that means expoſed by a very large ſurface to be carried off by the atmoſphere; and that the loſs of this watery vapour, which the flannel ſuſtains on the one ſide, by evaporation, being immediately reſtored from the other, in conſequence of the ſtrong attraction between the flannel and this vapour, the pores of the ſkin are diſencumbered, and they are continually ſurrounded by a dry, warm, and ſalubrious atmoſphere.

He is aſtoniſhed, that the cuſtom of wearing flannel next the ſkin ſhould not have prevailed more univerſally. He is confident it would prevent a multitude of diſeaſes; and he knows of no greater luxury than the comfortable ſenſation which arises from wearing it, especially after one is a little accustomed to it.

He considers it as a mistaken notion, that flannnel is too warm a cloathing for summer. He has worn it in the hottest climates, and in all seasons of the year, and never found the least inconvenience from it. He observes, that it is the warm bath of a perspiration confined by a linen shirt, wet with sweat, that renders the summer heats of southern climates so insupportable; but flannel, he adds, promotes perspiration, and favours its evoporation; and evaporation as it is well known, produces positive cold.

