# Errata

**Published:** 1788

**Authors:** 


					??2c6 for * evaporation' read 1 evaporation

				

